# Dr. Ferguson, on the Tongue and Its Diseases

**Published:** 1801-03

**Authors:** Andrew Ferguson

**Affiliations:** Aberdeen


### *To the Editors of the Medical and Phyſical Journal.*

GENTLEMEN,

IF the following Obſervations on the Tongue and its Diſeaſes are thought worthy a place in your truly valuable Publication, by inſerting them you will oblige your moſt obedient humble ſervant,

*Aberdeen, Jan.* 14, 1801. ANDREW FERGUSON.

---

The tongue is one of thoſe organs of the human body which is but very ſeldom affected with diſeaſes; and when they do occur, they ſeem to ariſe from ſomething of an infectious nature.

In my practice I have met with three different kinds of ulcerations upon the tongue, called aphtha, ſibbenic, and mercurial ulcers; theſe, I conſider as its moſt frequent morbid affections. Aphtha is a diſeaſe moſt commonly ſeizing children when they are very young; it is conſidered as one of the genus of the order exanthemata, accompanied with ſynochus fever, and little whitiſh ulcers, which are ſometimes diſtinct, at other times running together, ſpreading over the tongue and ſurface of the mouth, attended with pain, and ſlight ſwelling of the tongue; when the ſloughs are removed they ſoon grow again: the time of its going off is variable.

This is the definition of aphtha, and ſerves very well for diſtinguiſhing it; about its cauſes, however, there are various opinions entertained. Although ſeveral of the order of exanthemata are contagious, yet this diſeaſe is not mentioned as ſuch; it is, however, ſaid to be attended with ſynochus; and as moſt of the fevers of this ſpecies are contagious, if we reaſon from analogy, we muſt ſuſpect aphtha of being ſo too. I ſuppoſe that aphtha is rather at firſt a local affection, which has ſomething of a poiſonous ſtimulating nature attached to it, proceeding from a certain morbid condition of the glands of the mammæ: This ſtimulating ichor, iſſuing from the morbid glands, and mixing with the milk, I conſider as the cauſe of this ulceration of the child's tongue. Aphtha does not occur to every child, neither do I ſuppoſe ſuch a morbid ſtate common to the glands of every woman: In the firſt milk of every nurſe, there is ſomething of a ſtimulating nature, which acts upon the child as a cathartic. May not this ſtimulating quality be increaſed ſometimes, ſo as to occaſion this diſeaſe? I have obſerved before, that aphtha is at firſt of a local nature, but but when it happens to be violent, it is attended with fever, and universal affection: It descends by the primæ viæ, occasioning violent gripes, diarrhœa, and very often convulsions. This disease always infects the woman's nipples, producing sores very painful and difficult to heal, and which never will heal as long as the aphtha continues in the child's mouth. Are those who have been affected with aphtha, exempted from, or less liable to be affected with, the small-pox? I ask this question, because, having inoculated a daughter of Mr. Davidson, butcher, in this place, who was violently affected with aphtha when very young, she did not take the infection. Some time after, when I was inoculating four children of Mr. Alexander Sangster, a relation of Mr. D. Mrs. Davidson desired that I should, a second time, attempt to inoculate her daughter. The first time, her arm became inflamed a little, and a small pustule appeared; the second, as a larger wound was made, and a greater quantity of variolous matter introduced, the pustule was large, and attended with inflammation around; yet no eruption appeared upon the rest of her body: the other children who were inoculated with the same matter, had a good many very fine and distinct small-pox. Sibbenic ulcers proceed from a poison similar to syphilis, and are only a modification of it; the same agents which cure the one, cure the other. I have noticed this difference, however, that mercury has a more powerful effect in curing sibbens than syphilis; this I suppose to be owing to the difference of the stages of the complaint. I have observed, that in the first stage of syphilis, mercury in general has but very little effect until the poison begins to exert its influence upon the lymphatic system, and has suffered some change in the body. The sibbenic ulcers have a very different appearance from aphtha; they have a broader surface, appear principally upon the sides of the tongue; their surface is whitish, but their edges are swelled, and they look very much like cancerous sores: Nothing has so great an effect in removing these sores, as the topical application of muriated mercury, along with the agency of the mercurial pill. Mercurial ulcers are occasioned by mercury, applied either inwardly or outwardly; they want the cancerous appearance of the sibbenic ulcers; have a darker coloured surface in the mouth, and are attended with a disagreeable fœtid breath, and a taste resembling that of copper; unless when they are combined with the syphilitic poison, they require very little medicine, except the frequent application of emollient gargles, to which may be added, a small quantity of the sulphuret of potash.

The tongue is less liable to be affected with inflammation than any of the other organs of the human body, and yet it is more more expofed than any of them to the action of ftimuli, both of the folid and fluid kind; this muft be owing to the ftructure of its external covering, which feems to be peculiar to itfelf. The internal fubftance of the tongue is compofed of feveral mufcles, called genio-gloffus, cerato-gloffus, ftylo-gloffus, and lingualis; the fibres of which are difpofed in a longitudinal tranfverfe, and vertical manner; they enable the tongue to move in all directions, which is important in the operation of fuction, maftication, deglutition, and the articulation of the voice: A defect of the mufcles of the tongue produces paraphonia, and feveral of the fpecies of pfellifmus. One of the principal ufes of the tongue is, to afford the fenfe of tafte; for this purpofe it is covered with numerous papillæ, which, from their appearance, have been diftinguifhed by the names of capitatæ, femi-lenticulares, and villofæ; thefe have a communication with the nerves of the tongue: The capitatæ appear principally upon the bafis of the tongue. I have known more than half a dozen of thefe in feveral perfon's tongues to be enlarged to the fize of fmall peas, without their being even fenfible of it; the great variety of taftes is occafioned by the action of the ftimulus of our food, &c. on the papillæ. Some animals, owing to the particular ftructure of thefe, have the fenfe of tafte more acutely than the human fpecies. When the papillæ are difeafed, or hurt by the repeated action of violent ftimuli and narcotics, (fuch as opium and tobacco) their fenfibility is much diminifhed, and the tafte impaired. In peripneumony and catarrh, I have obferved that the fenfe of tafte is very much impaired; and when this is the cafe, I have found it to be a very good fign.

In every univerfal difeafe, I make it a common rule to caufe the patient to fhow me his tongue, and inquire concerning his fenfe of tafte; this I confider to be very material, when prefcribing, as fometimes his acutenefs of tafte might prevent him from taking certain very efficacious remedies, which often prove more difagreeable to the tongue than to the ftomach. Inability to thruft out the tongue, and lofs of tafte, have been confidered as prognoftic figns, indicating great debility and danger: Thefe moft readily occur in the latter ftages of typhus. In the beginning of fever, when the debility of the functions is not fo extreme as in the after ftages, we often perceive a tremor of the tongue, when it is thruft out: We can form very little idea of the ftate of the ftomach from the appearance of the tongue. What we term a *fur* upon the tongue, is not a certain fign that the ftomach is foul. I agree with Dr. Domier in this particular. We often find the tongue foul when the digeftion is good, and *vice verfa.* Some perfons have this *fur* upon their tongues at all times, even from the apex to the bafe; others have very clean tongues, who are much troubled with dyfpepfia. I am acquainted with a perfon whofe tongue is full of hacks and deep furrows, which interfect it in all directions, and a very deep one in the courfe of the linea linguæ mediana: His tongue is feldom white or foul, but appears of a bright red colour, and he has no other complaint excepting a few rotten teeth.

In no univerfal difeafe is the tongue fo much affected as in fevers, efpecially when they are long protracted, or attended with putrid fymptoms. In typhus and its varieties, from the very beginning the tongue is *furred*. As the fever advances, it turns to an afh-coloured cruft, which afterwards changes to a brown. The fame appearance of the tongue accompanies feveral other difeafes, fuch as cynanche maligna, tonfillaris, parotidœa, and fcarlatina anginofa. In all difeafes where there is great debility, attended with putrefcency, the tongue is moft affected with *fur*. The caufe of this appearance muft be debility, and a relaxed ftate of the furface of the tongue and throat, from a deficiency of excitement; for I have frequently obferved, that thofe who are of a florid colour, and healthy appearance, have generally the furface of their tongue of a redder colour, and cleaner than thofe who are weakly and pale. The tongue is always of a more beautiful colour at its extremity than its bafe; and when cruft, or *fur*, of any kind appears, the greateft quantity is always at the root of the tongue, where there is generally the leaft motion. In the morning, after the tongue has been at reft all night, it always looks fouleft: Nothing ferves the purpofe of cleaning it fo well as its action in maftication, and frequent fpeaking, which promotes the circulation of the faliva, and removes every collection of foulnefs that gathers in the mouth. If this fhould be found ineffectual, recourfe may be had to tonic gargles, as the beft means of removing *fur*, and preferving a vigorous ftate of the papillæ. The tongue, at its anterior and under part, has a ligamentous band, called frænum, formed of a reduplication of the membrane that lines the infide of the mouth; this feems for fixing and preventing it from making too much motion. When the frænum happens to be too near the extremity of the tongue, fo that it becomes fixed to that degree that the child cannot put it out, or ufe it, then the difeafe, known by the name of *tongue-tied*, takes place. There may be feveral degrees of this; that which requires manual affiftance does not fo often occur as is generally imagined, and many times the operation is performed by midwifes when there is no real neceffity for it.

